# Effect of *BRAF* mutation on the prognosis for patients with colorectal cancer undergoing cytoreductive surgery for synchronous peritoneal metastasis

**DOI:** 10.1093/gastro/goad061

**Published:** 2023-10-24

**Authors:** Zhijie Wu, Xiusen Qin, Yuanxin Zhang, Jian Luo, Rui Luo, Zonglu Cai, Hui Wang

**Affiliations:** Department of General Surgery (Colorectal Surgery), The Sixth Affiliated Hospital, Sun Yat-sen University, Guangzhou, Guangdong, P. R. China; Guangdong Provincial Key Laboratory of Colorectal and Pelvic Floor Diseases, The Sixth Affiliated Hospital, Sun Yat-sen University, Guangzhou, Guangdong, P. R. China; Biomedical Innovation Center, The Sixth Affiliated Hospital, Sun Yat-sen University, Guangzhou, Guangdong, P. R. China; Department of General Surgery (Colorectal Surgery), The Sixth Affiliated Hospital, Sun Yat-sen University, Guangzhou, Guangdong, P. R. China; Guangdong Provincial Key Laboratory of Colorectal and Pelvic Floor Diseases, The Sixth Affiliated Hospital, Sun Yat-sen University, Guangzhou, Guangdong, P. R. China; Biomedical Innovation Center, The Sixth Affiliated Hospital, Sun Yat-sen University, Guangzhou, Guangdong, P. R. China; Department of General Surgery (Colorectal Surgery), The Sixth Affiliated Hospital, Sun Yat-sen University, Guangzhou, Guangdong, P. R. China; Guangdong Provincial Key Laboratory of Colorectal and Pelvic Floor Diseases, The Sixth Affiliated Hospital, Sun Yat-sen University, Guangzhou, Guangdong, P. R. China; Biomedical Innovation Center, The Sixth Affiliated Hospital, Sun Yat-sen University, Guangzhou, Guangdong, P. R. China; Department of General Surgery (Colorectal Surgery), The Sixth Affiliated Hospital, Sun Yat-sen University, Guangzhou, Guangdong, P. R. China; Guangdong Provincial Key Laboratory of Colorectal and Pelvic Floor Diseases, The Sixth Affiliated Hospital, Sun Yat-sen University, Guangzhou, Guangdong, P. R. China; Biomedical Innovation Center, The Sixth Affiliated Hospital, Sun Yat-sen University, Guangzhou, Guangdong, P. R. China; Department of General Surgery (Colorectal Surgery), The Sixth Affiliated Hospital, Sun Yat-sen University, Guangzhou, Guangdong, P. R. China; Guangdong Provincial Key Laboratory of Colorectal and Pelvic Floor Diseases, The Sixth Affiliated Hospital, Sun Yat-sen University, Guangzhou, Guangdong, P. R. China; Biomedical Innovation Center, The Sixth Affiliated Hospital, Sun Yat-sen University, Guangzhou, Guangdong, P. R. China; Department of General Surgery (Colorectal Surgery), The Sixth Affiliated Hospital, Sun Yat-sen University, Guangzhou, Guangdong, P. R. China; Guangdong Provincial Key Laboratory of Colorectal and Pelvic Floor Diseases, The Sixth Affiliated Hospital, Sun Yat-sen University, Guangzhou, Guangdong, P. R. China; Biomedical Innovation Center, The Sixth Affiliated Hospital, Sun Yat-sen University, Guangzhou, Guangdong, P. R. China; Department of General Surgery (Colorectal Surgery), The Sixth Affiliated Hospital, Sun Yat-sen University, Guangzhou, Guangdong, P. R. China; Guangdong Provincial Key Laboratory of Colorectal and Pelvic Floor Diseases, The Sixth Affiliated Hospital, Sun Yat-sen University, Guangzhou, Guangdong, P. R. China; Biomedical Innovation Center, The Sixth Affiliated Hospital, Sun Yat-sen University, Guangzhou, Guangdong, P. R. China

**Keywords:** colorectal cancer, peritoneal metastasis, *BRAF*, prognosis

## Abstract

**Background:**

*KRAS/BRAF* mutations (mut*KRAS/*mut*BRAF*) are unfavorable prognostic factors for colorectal cancer (CRC) metastases to the liver and lungs. However, their effects on the prognosis for patients with synchronous peritoneal metastasis (S-PM) of CRC after cytoreductive surgery (CRS) and hyperthermic intraperitoneal chemotherapy (HIPEC) are controversial. In the study, we aimed to determine the effects of mut*KRAS/*mut*BRAF* on the prognosis for patients with S-PM who received CRS.

**Methods:**

A total of 142 patients diagnosed with S-PM between July 2007 and July 2019 were included in this study. The demographics, mut*KRAS/*mut*BRAF* status, overall survival (OS), and progression-free survival (PFS) of the patients were evaluated. The Kaplan–Meier method and log-rank test were used to estimate the difference in survival between groups.

**Results:**

Among 142 patients, 68 (47.9%) showed mut*KRAS* and 42 (29.5%) showed mut*BRAF*. The median OS values were 8.4 and 34.3 months for patients with mut*BRAF* and *BRAF* wild-type, respectively (*P *<* *0.01). However, *KRAS* status was not significantly associated with median OS (*P *=* *0.76). Multivariate analysis revealed carcinoembryonic antigen, CRS, HIPEC, and mut*BRAF* as independent predictors for OS. Based on these findings, a nomogram was constructed. The C-index was 0.789 (95% confidence interval, 0.742–0.836), indicating good predictive ability of the model. Furthermore, the 1- and 2-year survival calibration plots showed good agreement between the predicted and actual OS rates. The area under curves of the 1- and 2-year survival predictions based on the nomogram were 0.807 and 0.682, respectively. Additionally, mut*BRAF* was significantly associated with lower PFS (*P *<* *0.001).

**Conclusions:**

mut*BRAF* is an independent prognostic risk factor for S-PM. The established nomogram predicted the OS of patients with CRC having S-PM with high accuracy, indicating its usefulness as a valuable prognostic tool for the designated patient cohort.

## Introduction

Colorectal cancer (CRC) is the third most common malignant tumor worldwide, with tumor metastasis and recurrence being the leading cause of death among these patients [1]. The most common site of metastasis in patients with CRC is the liver, accounting for 60% of patients with metastatic CRC (mCRC); peritoneal metastasis (PM) accounts for ∼20% of patients with mCRC [2, 3]. Jayne *et al*. [4] reported that 5%–10% of newly diagnosed patients with CRC presented with PM, which was referred to as synchronous PM (S-PM). The prognosis for PM was worse than that of isolated distant metastasis at other sites [5]. Moreover, the median overall survival (mOS) was 16.3 months for patients with isolated PM and 24.6 months for those with isolated lung metastasis [6].

Cytoreductive surgery (CRS) with hyperthermic intraperitoneal chemotherapy (HIPEC) is the best treatment option for patients with PM because it may result in long-term survival [7, 8]. Macroscopic tumors in the abdominal cavity are resected in CRS and residual tumors in the abdominal cavity may be killed by the synergistic effect of hyperthermia and chemotherapy in HIPEC. Previous studies have shown that for patients with PM who have undergone CRS with HIPEC, the 5-year survival rate was as high as 40%, with an mOS of ∼40 months [9, 10].

The Ras/Raf/mitogen-activated protein kinase signaling pathway plays a vital role in CRC carcinogenesis [10, 11]. *KRAS* mutation (mut*KRAS*) and *BRAF* mutation (mut*BRAF*) occur in ∼40% and ∼20% of patients with mCRC, respectively [11, 12]. These mutations are considered unfavorable prognostic factors for liver and lung metastases in patients with CRC [13–15]. Nevertheless, the effects of mut*KRAS* and mut*BRAF* on the prognosis for patients with CRC and PM remain controversial. A study has shown that mut*KRAS* and mut*BRAF* negatively affect PM [16]. However, other studies have indicated that only mut*BRAF* is associated with patient prognosis and that the decision for surgery in such patients should be taken with caution [17–19]. Furthermore, no studies have been performed to investigate the effects of mut*KRAS* and mut*BRAF* on the prognosis for patients with CRC and S-PM.

In the present study, we determined the effects of mut*KRAS* and mut*BRAF* on the prognosis for patients with CRC showing S-PM who underwent CRS. Furthermore, we developed and validated an innovative prognostic nomogram for the mentioned patient cohort.

## Patients and methods

### Patient selection

Between July 2007 and July 2019, 653 patients were diagnosed with PM at the Sixth Affiliated Hospital, Sun Yat-sen University (Guangzhou, China). We included patients with pathologically confirmed CRC and PM but without liver and lung metastases in this study. The exclusion criteria were as follows: (i) patients without complete clinicopathological and follow-up data; (ii) those who did not undergo CRS; or (iii) those with confirmed CRC and metachronous PM.

### Treatment

A multidisciplinary team examined all patients before the surgery. CRS included the resection of primary and invasive peritoneal and visceral tumors as well as complete removal of tumor deposits in the abdominal cavity. Multiorgan resection was performed to achieve complete cytoreduction whenever necessary. Residual lesions were assessed on the basis of the completeness of cytoreduction (CC) score: CC-0, no visible peritoneal tumors after CRS; CC-1, presence of tumor nodules of size <2.5 mm; CC-2, presence of residual tumors of size 2.5–25 mm; and CC-3, presence of tumor nodules of size >25 mm or unresectable tumor nodules anywhere in the abdomen or pelvis. Because the operation involved the resection and anastomosis of the bowel, some patients who underwent CRS received preventive stoma care post-operatively. Furthermore, some patients received HIPEC post-operatively one to three times in a closed manner to kill free tumor cells and prevent tumor recurrence. Chemotherapeutic drugs such as 5-fluorouracil, oxaliplatin, and platinum were mixed with normal saline and heated to 42°C. The perfusion rate was maintained at 100 mL/min during initiation; the temperature of the perfusion fluid was maintained at 42°C for 1 h. The perioperative chemotherapy regimens, comprising mainly 5-fluorouracil, included FOLFOX, FOLFIRI, and XELOX.

### Clinicopathological characteristics and follow-up

The clinicopathological characteristics such as age, sex, T category, N category, peritoneal cancer index (PCI), CRS, HIPEC, CC score, preventive stoma, perioperative chemotherapy, tumor location, mut*KRAS* and mut*BRAF* status, and histology were considered during the study. The last follow-up was conducted on 20 June 2021. The primary end point was OS, which was defined as the period from the date of initial treatment (chemotherapy or surgical intervention) to the date of death or the last follow-up. The secondary end point was progression-free survival (PFS), which was defined as the time from initial treatment to tumor progression or death because of tumor progression or the last follow-up. The survival curve was plotted by using the Kaplan–Meier method. Univariate analysis was performed to identify prognostic factors associated with OS and PFS. Factors with a *P*-value of <0.05 were selected for multivariate Cox regression analysis.

### Nomogram development and validation

Multivariate Cox regression analysis was performed on variables with a *P*-value of <0.05 to determine independent prognostic factors. Further, a nomogram was established based on these variables to predict the 1- and 2-year OS in patients with S-PM. The C-index was used to evaluate the predictive accuracy of the nomogram model. Generally, the C-index ranges between 0.5 and 1, with 0.5 being completely random and 1 being completely predictable. Furthermore, a calibration plot was constructed by comparing the 1- and 2-year predicted and actual survival rates. Finally, the predictive performance of the nomogram model was verified by plotting a receiver-operating characteristic (ROC) curve.

### Statistical analysis

SPSS (Windows version 25.0) and R (version 4.0.3) software were used for statistical analysis. The R packages “survival,” “rms,” “foreign,” and “survivalROC” were used to establish the prognostic nomogram, calculate the C-index, and plot the calibration and ROC curves, respectively. The chi-square or Fischer’s exact test was performed to compare differences between the categorical variables. The Kaplan–Meier method and log-rank test were used to estimate the difference in OS and PFS. A *P*-value of <0.05 was considered statistically significant.

## Results

### Patients’ characteristics and OS

**Figure 1.**
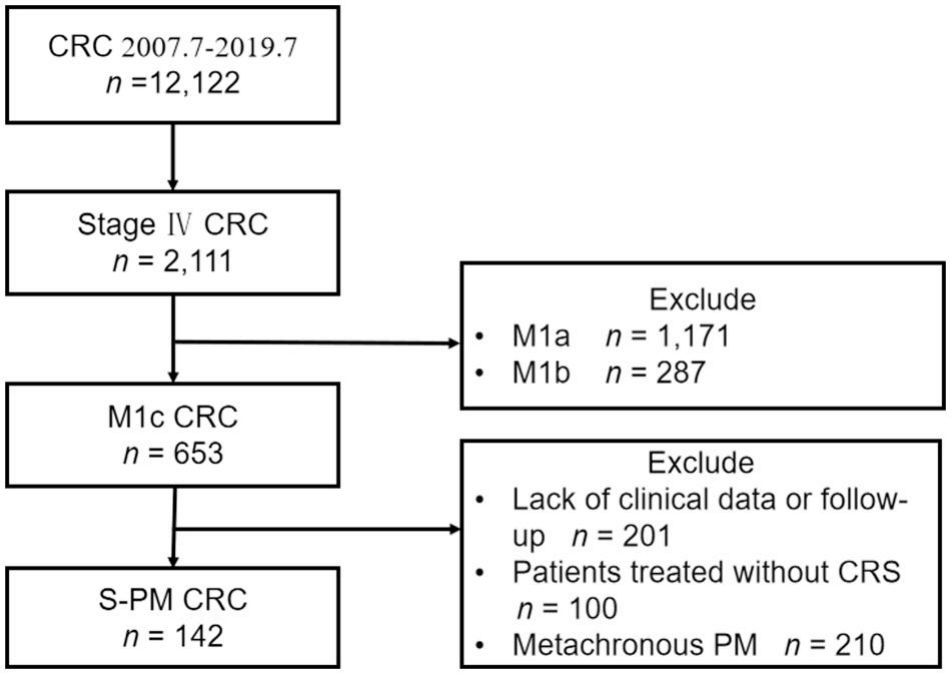
Flow chart of patient selection. CRC = colorectal cancer, S-PM = synchronous peritoneal metastasis, CRS = cytoreductive surgery.

Based on the inclusion and exclusion criteria, 142 patients pathologically diagnosed with CRC and S-PM were included (Figure 1). All patients underwent CRS; 55 patients (38.7%) had a CC score of 0/1 and 87 (61.3%) had a CC score of 2. In addition, 63 patients (44.3%) received HIPEC. Table 1 presents the clinicopathological characteristics and treatment details of the patients. The mOS of all patients was 23.8 months; the 1- and 2-year OS rates were 67.2% and 49.2%, respectively.

**Table 1.** Characteristics of the patients with CRC-SPM

Variable	No. of patients (%)
**Age (years)**	
≥60	59 (41.5)
<60	83 (58.5)
**Sex**	
Male	76 (53.5)
Female	66 (46.5)
**T category**	
T0–3	65 (45.7)
T4	77 (54.3)
**N category**	
N0	23 (16.1)
N1–2	119 (83.9)
**Location of tumor**	
Right-sided colon	54 (38.1)
Left-sided colon	69 (48.5)
Rectum	19 (13.4)
**Obstruction**	
Yes	85 (59.8)
No	57 (40.2)
**PCI**	
≥20	17 (11.9)
<20	125 (88.1)
**Histology**	
Adenocarcinoma	102 (71.9)
Mucinous adenocarcinoma and signet ring cell carcinoma	40 (28.1)
**CRS**	
CC-0/1	55 (38.7)
CC-2	87 (61.3)
**HIPEC**	
Yes	63 (44.4)
No	79 (55.6)
**Perioperative chemotherapy**	
Yes	98 (69.1)
No	44 (30.9)
**Preventive stoma**	
Yes	42 (29.6)
No	100 (70.4)
***KRAS* status**	
mut*KRAS*	68 (47.9)
wt*KRAS*	74 (52.1)
***BRAF* status**	
mut*BRAF*	42 (29.5)
wt*BRAF*	100 (70.5)

CRS = cytoreductive surgery, CC = completeness of cytoreduction, HIPEC = hyperthermic intraperitoneal chemotherapy, mut*KRAS*/wt*KRAS* = *KRAS* mutation/*KRAS* wild-type, mut*BRAF*/wt*BRAF* = *BRAF* mutation/*BRAF* wild-type.

### KRAS/BRAF analysis

Among 142 patients, 68 (47.9%) had mut*KRAS*, 42 (29.5%) had mut*BRAF*, and 58 (40.8%) possessed a dual wild-type (WT) genotype. mut*KRAS* tumors were evenly distributed in the left and right colon and the rectum, whereas mut*BRAF* tumors were mainly located in the right colon (*P *=* *0.060) (Table 2). The mut*BRAF* and *BRAF* WT (wt*BRAF*) groups showed significant differences in the T category, post-operative HIPEC, and tumor location; no significant differences were observed between the mut*KRAS* and *KRAS* WT (wt*KRAS*) groups.

**Table 2.** Summary of the demographics of the patients treated with CRS according to the *KRAS*/*BRAF* status

Variable	wt*BRAF* (*n *=* *100)	mut*BRAF* (*n *=* *42)	*P*	wt*KRAS* (*n *=* *74)	mut*KRAS* (*n *=* *68)	*P*
**Age (years)**			0.190			0.106
≥60	38 (38.0)	21 (50.0)		26 (35.1)	33 (48.5)	
<60	62 (62.0)	21 (50.0)		48 (64.9)	35 (51.5)	
**Sex**			0.200			0.639
Male	57 (57.0)	19 (45.2)		41 (55.4)	35 (51.5)	
Female	43 (43.0)	23 (54.8)		33 (44.6)	33 (48.5)	
**T category**			0.002			0.768
T0–3	54 (54.0)	11 (26.2)		33 (44.6)	32 (47.1)	
T4	46 (46.0)	31 (73.8)		41 (55.4)	36 (52.9)	
**N category**			0.680			0.653
N0	17 (17.0)	6 (14.3)		11 (14.9)	12 (17.6)	
N1–2	83 (83.0)	36 (85.7)		63 (85.1)	56 (82.4)	
**CRS**			0.180			0.819
CC-0/1	43 (43.0)	12 (28.5)		28 (37.9)	27 (39.7)	
CC-2	57 (57.0)	30 (71.5)		46 (62.1)	41 (60.3)	
**HIPEC**			0.030			0.695
Yes	50 (50.0)	13 (31.0)		36 (48.6)	27 (39.7)	
No	50 (50.0)	29 (69.0)		38 (51.4)	41 (60.3)	
**Perioperative chemotherapy**			0.240			0.452
Yes	72 (72.0)	26 (62.0)		49 (66.2)	49 (72.1)	
No	28 (28.0)	16 (38.0)		25 (33.8)	19 (27.9)	
**Preventive stoma**			0.820			0.682
Yes	29 (29.0)	13 (31.0)		23 (31.1)	19 (27.9)	
No	71 (71.0)	29 (69.0)		51 (68.9)	49 (72.1)	
**Location of tumor**			0.060			0.320
Right-sided colon	43 (71.0)	26 (62.0)		33 (44.6)	36 (52.9)	
Left-sided colon	43 (43.0)	11 (26.2)		30 (40.5)	24 (35.3)	
Rectum	14 (14.0)	5 (11.8)		11 (14.9)	8 (11.8)	
**PCI**			0.980			0.040
≥20	12 (12.0)	5 (11.8)		5 (6.8)	12 (17.6)	
<20	88 (88.0)	37 (88.1)		69 (93.2)	56 (82.4)	
**Histology**			0.630			0.950
Adenocarcinoma	73 (73.0)	29 (69.0)		53 (71.6)	49 (72.1)	
Mucinous adenocarcinoma and signet ring cell carcinoma	27 (27.0)	13 (31.0)		21 (28.4)	19 (27.9)	

All values are presented as number of patients followed by percentage in the parentheses.

CRS = cytoreductive surgery, HIPEC = hyperthermic intraperitoneal chemotherapy, mut*KRAS*/wt*KRAS* = *KRAS* mutation/*KRAS* wild-type, mut*BRAF*/wt*BRAF* = *BRAF* mutation/*BRAF* wild-type.

**Figure 2.**
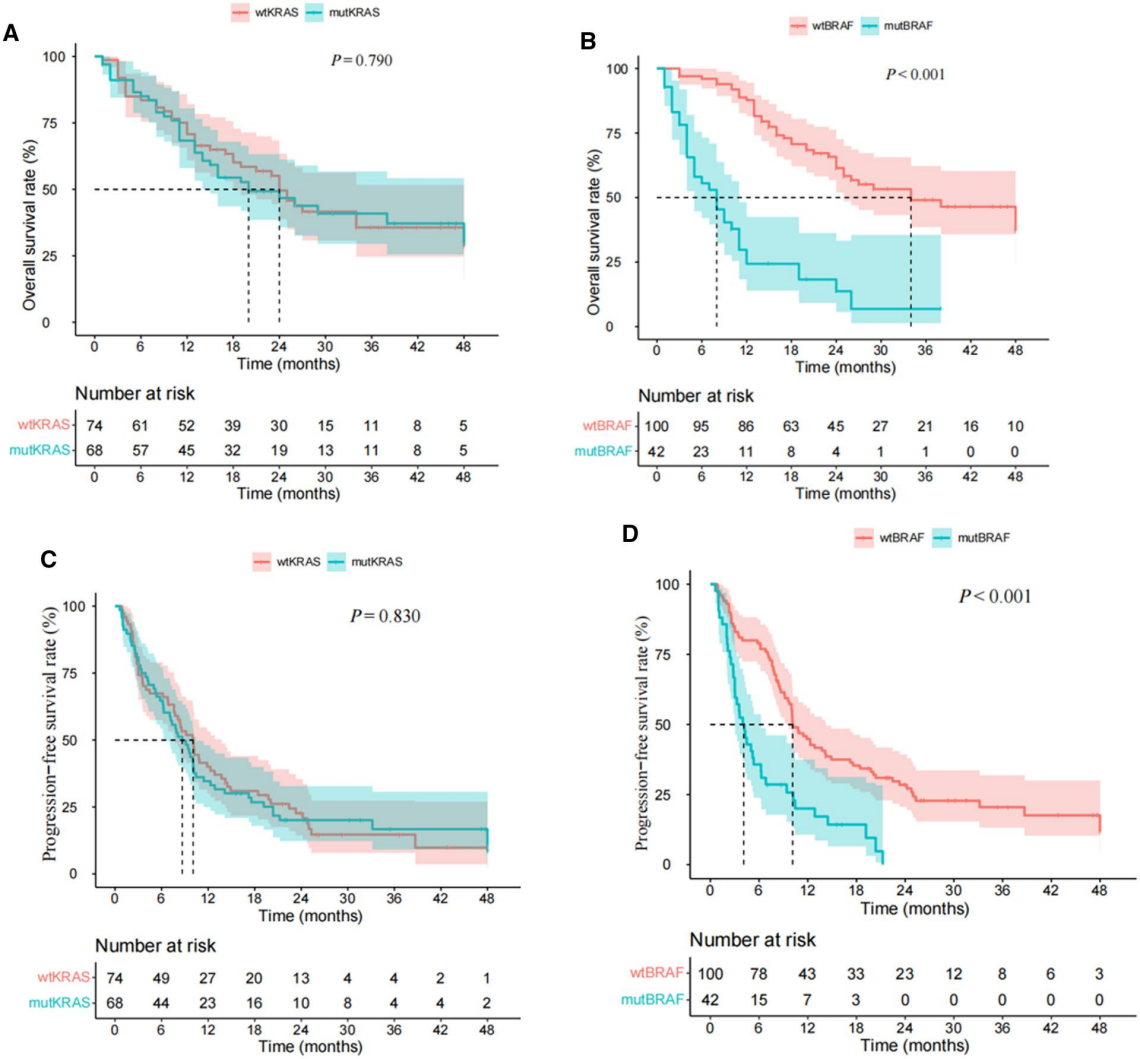
The Kaplan–Meier curve of overall survival and progression-free survival for patients with different *KRAS* and *BRAF* status*.* (A) The Kaplan–Meier curve of overall survival for patients with mut*KRAS* and wt*KRAS.* (B) The Kaplan–Meier curve of overall survival for patients with mut*BRAF* and wt*BRAF*. (C) The Kaplan–Meier curve of progression-free survival for patients with mut*KRAS* and wt*KRAS.* (D) The Kaplan–Meier curve of progression-free survival for patients with mut*BRAF* and wt*BRAF*. mut*KRAS*/wt*KRAS* = *KRAS* mutation/*KRAS* wild-type, mut*BRAF*/wt*BRAF* = *BRAF* mutation/*BRAF* wild-type.

The mOS values were 20.2 and 24.1 months for patients with mut*KRAS* and wt*KRAS*, respectively (Figure 2A). No significant difference in the prognosis was observed between the two groups (*P *=* *0.760). The mOS values were 8.4 and 34.3 months for patients with mut*BRAF* and wt*BRAF*, respectively (Figure 2B). In contrast to the results for *KRAS*, patients with mut*BRAF* had a worse prognosis (*P *<* *0.001).

The median PFS for all patients was 9.8 months. The median PFS values were 8.8 and 10.4 months for patients with mut*KRAS* and wt*KRAS*, respectively (Figure 2C). No significant difference in the prognosis was observed between the two groups (*P *=* *0.830). The median PFS values were 4.2 and 10.2 months for patients with mut*BRAF* and wt*BRAF*, respectively (Figure 2D). In contrast to the results for *KRAS*, patients with mut*BRAF* had a worse prognosis (*P *<* *0.001).

### Univariate and multivariate Cox analyses

The following factors associated with better outcomes in patients with S-PM CRC were identified by performing univariate Cox analysis: carcinoma embryonic antigen (CEA) of <5.0 ng/mL (*P *=* *0.008), CA199 of <37.0 U/mL (*P *=* *0.008), CC-0/1 (*P *<* *0.001), HIPEC (*P *=* *0.001), and wt*BRAF* (*P *<* *0.001) (Table 3). The multivariate Cox analysis further revealed CEA of <5.0 ng/mL, CC-0/1, HIPEC, and wt*BRAF* as independent prognostic factors for OS (Table 3). Thus, these factors were used to construct a nomogram.

**Table 3.** Cox univariate and multivariate analyses of the 142 patients with S-PM CRC

Variable	Univariate analysis		Multivariate analysis	
	HR (95% CI)	*P*	HR (95% CI)	*P*
Sex (female)	1.29 (0.83–2.02)	0.250		
CEA (≥5.0 ng/mL)	1.91 (1.18–3.09)	0.008	1.66 (1.01–2.73)	0.040
CA199 (≥37.0 U/mL)	1.82 (1.17–2.86)	0.008	1.19 (0.74–1.91)	0.450
CA125 (≥35.0 U/mL)	1.22 (0.88–1.62)	0.350		
Location				
Right-sided colon	Reference	0.570		
Left-sided colon	0.51 (0.31–0.83)			
Rectum	0.83 (0.44–1.58)			
Obstruction	1.43 (0.89–2.29)	0.130		
CRS (CC-2)	3.49 (2.02–6.01)	<0.001	2.81 (1.59–4.99)	<0.001
HIPEC (Yes)	0.46 (0.29–0.73)	0.001	0.53 (0.32–0.86)	0.010
*KRAS* (mut)	1.06 (0.68–1.65)	0.790	–	
*BRAF* (mut)	4.32 (2.72–6.85)	<0.001	4.35 (2.69–7.01)	<0.001
Category (T4)	1.52 (0.97–2.38)	0.060		
Category (N1–2)	1.14 (0.82–1.59)	0.420		
Histology (mucinous adenocarcinoma and signet ring cell carcinoma)	1.53 (0.92–2.53)	0.090		
PCI (≥20)	1.21 (0.62–2.35)	0.600		

CRC = colorectal cancer, S-PM = synchronous peritoneal metastasis, CRS = cytoreductive surgery, HIPEC = hyperthermic intraperitoneal chemotherapy, mut*KRAS*/wt*KRAS* = *KRAS* mutation/*KRAS* wild-type, mut*BRAF*/wt*BRAF* = *BRAF* mutation/*BRAF* wild-type.

### Nomogram construction and validation

**Figure 3.**
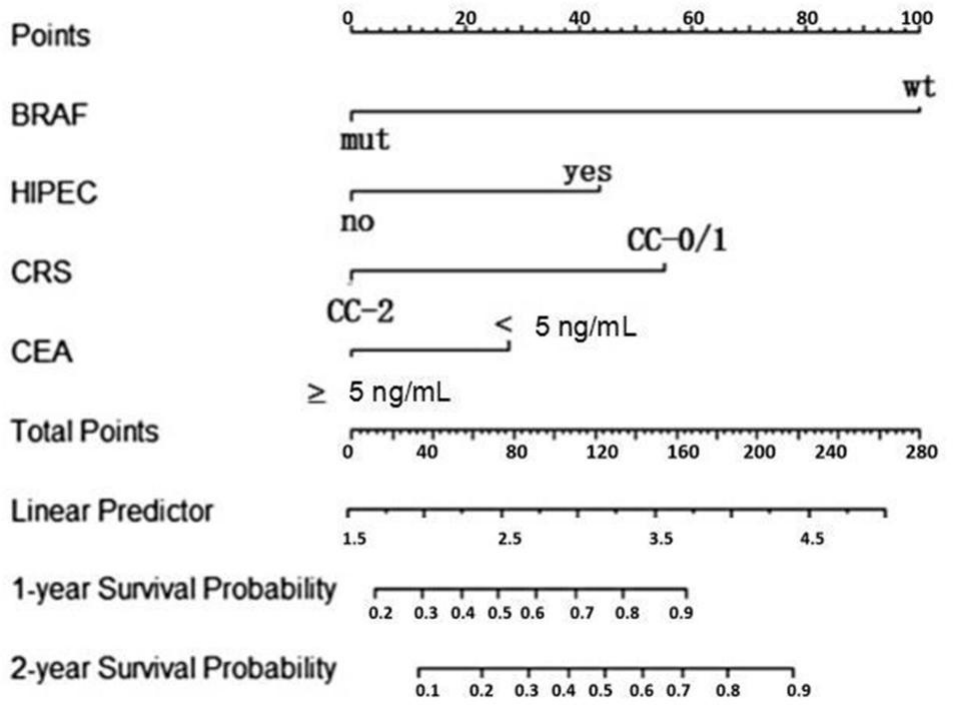
The nomogram of predicting the 1- and 2-year post-operative survival probabilities of patients with synchronous peritoneal metastasis. mut = mutant, wt = wild-type, HIPEC = hyperthermic intraperitoneal chemotherapy, CRS = cytoreductive surgery, CC = completeness of cytoreduction, CEA = carcinoma embryonic antigen.

**Figure 4.**
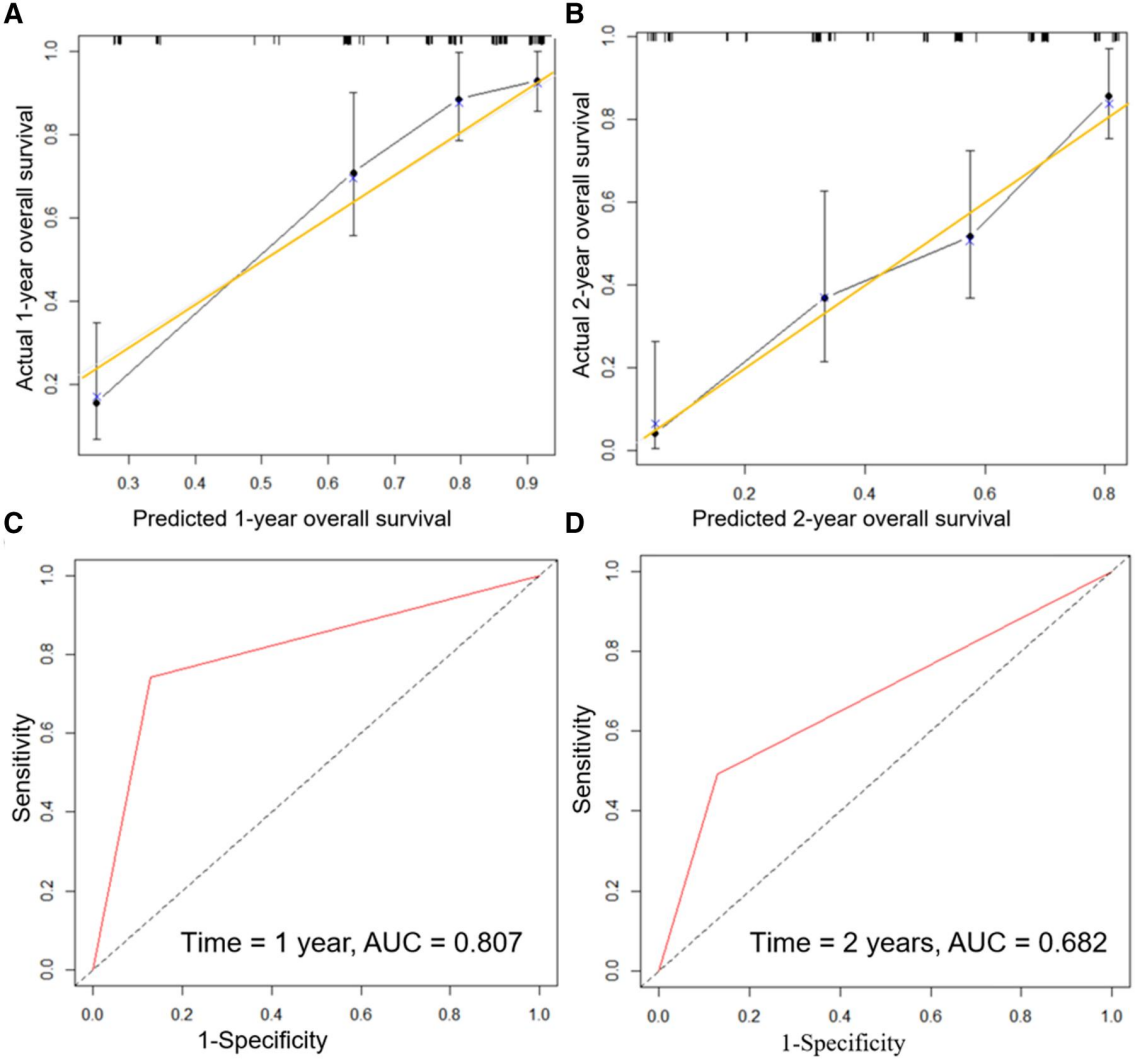
The calibration plots and ROC curve of the nomogram. (A) and (B) The 1- and 2-year calibration plots of the nomogram. The horizontal axes display the nomogram-predicted probabilities of overall survival at 1 and 2 years, whereas the vertical axes display the actual overall survival rates at 1 and 2 years. The diagonal line from the lower left to the upper right corner of the plot area is a reference line indicating the ideal prediction. The predictive accuracy of the model was assessed by using an ROC curve. (C) and (D) The AUC values for the 1- and 2-year survival predictions of the nomogram. The larger the AUC value, the better the prediction performance of the nomogram model. ROC curve = receiver-operating characteristic curve, AUC = area under the curve.

The 1- and 2-year post-operative survival probabilities for patients with S-PM were predicted using the constructed nomogram (Figure 3). To evaluate nomogram performance, we used the C-index to evaluate the ability of the nomogram to predict the 1- and 2-year OS of the patients. The C-index was 0.789 (95% confidence interval [CI], 0.742–0.836), which indicated the good prediction ability of the nomogram model. Furthermore, the 1- and 2-year calibration plots showed good agreement between the predicted and actual survival rates in the training cohort (Figure 4A and B). The area under the curve values for the 1- and 2-year survival predictions on the basis of the constructed nomogram were 0.807 and 0.682, respectively (Figure 4C and D).

## Discussion

The present retrospective analysis of 142 patients with S-PM admitted at our hospital showed that the mut*KRAS* and mut*BRAF* rates were 47.9% (68/142) and 29.5% (42/142), respectively. mut*BRAF* was identified as an independent risk factor for OS and PFS, but mut*KRAS* was not. Furthermore, CEA, CRS, and HIPEC were identified as independent prognostic factors for OS.

Current guidelines and relevant studies have confirmed that CRS, HIPEC, and systemic chemotherapy can considerably improve survival in selective patients with S-PM CRC [9, 20–22]. We found that patients who underwent CRS and received HIPEC had a good survival prognosis (mOS, 30.2 months). However, whether HIPEC is beneficial for patients with PM CRC remains controversial. The PRODIGE 7 trial showed that, compared with CRS alone, CRS + HIPEC provided no additional survival benefits for patients with PM [23]. However, the trial did not distinguish the survival benefits between metachronous and synchronous colorectal PMs. All patients included in the present study showed S-PM. Patients with metachronous PM are more prone to relapse after HIPEC than those with S-PM [24].

CC and PCI scores are the key factors affecting the prognosis for patients who undergo CRS [25, 26]. Herein, CC-0/1 was identified as a prognostic factor for patients who underwent CRS. Sugarbaker [27] reported that the 5-year survival rate for patients with CRC peritoneal metastases with PCI of >20 was 0 and suggested that such patients should receive palliative care. Thus, some researchers consider a PCI of ≤19 as the cut-off value [28, 29]. According to different primary tumor types, some researchers consider a PCI of ≤15 or 17 as the cut-off values for PM in gastric cancer [30, 31]. Nonetheless, the PCI cut-off value remains controversial worldwide. Herein, a PCI of >20 was not significantly associated with patient prognosis, which might be due to the low PCI score (the patients with PCI > 20 accounted for 11.9% of all; the mean PCI score was 6.2 ± 7.4).

Studies have shown mut*KRAS* and mut*BRAF* occur in ∼40% and ∼5%–20% of patients with mCRC, respectively [14, 32]. The incidence rates of mut*KRAS* and mut*BRAF* in PM CRC have been reported to be 45% and 24%, respectively [16, 18]. In the present study, the occurrence rates of mut*KRAS* and mut*BRAF* were slightly higher, reaching 47.9% and 29.5%, respectively. The possible reason is that these patients had S-PM. Moreover, 38.1% of the patients showed right-sided primary tumors, 48.2% showed left-sided tumors, and 13.4% showed rectal tumors. A previous study showed a higher rate of mut*BRAF* in the right-sided colon than in other sites in patients with CRC [33]. However, we could not determine the relationship between mut*KRAS*/mut*BRAF* and tumor location in the present study.

The prognosis for patients with mCRC showing liver and lung metastases is inferior to that for patients with peritoneal metastases [6]. mut*KRAS* and mut*BRAF* are unfavorable prognostic factors in patients with CRC showing liver and lung metastases [13, 14]. However, the association of these mutations with PM in patients with CRC remains controversial. Tonello *et al*. [16] found that, compared with patients harboring WT mutations, those with mut*KRAS* and mut*BRAF* exhibited shorter 5-year OS (29.4% and 26.8% vs 51.5%, respectively). Morgan *et al*. [19] found that mut*KRAS* was significantly associated with decreased recurrence-free survival, but not with OS. Conversely, Graf *et al*. [34] found no difference in OS between patients carrying mut*KRAS* and those with wt*KRAS*. Furthermore, Larsen *et al*. [18] found that the OS rates of patients with mut*BRAF*, mut*KRAS*, and double-WT were similar. The present study showed mut*BRAF* as a prognostic risk factor for patients who underwent CRS, whereas mut*KRAS* was not considered a prognostic factor. In Tonello *et al*.’s [16] and Larsen *et al*.’s [18] studies, patients with S-PM and metachronous PM (M-PM) were included. The proportion of patients with S-PM and M-PM accounted for 71.8% and 66.1%, respectively. However, all 142 patients in the present study had S-PM, whereas Morgan *et al*.’s [19] study included 45 patients with M-PM. Graf *et al*. [34] included patients with appendiceal primaries and those who received palliative care. These differences in patient conditions may have contributed to the differences in the results.

Studies on primary CRC have shown that the mismatch repair status is crucial in explaining the mut*BRAF* status, which did not affect OS and disease-free survival in patients with microsatellite instability (MSI) tumors [35, 36]. MSI and mut*BRAF* are independent adverse prognostic factors for mCRC [36]. Sherman *et al*. [37] found that, compared with patients with microsatellite stability, patients with PM + MSI showed worse survival. Owing to the retrospective nature of the present study, the mismatch repair status of all patients could not be detected. Furthermore, the data of some patients were missing; thus, the complete data of only 12 patients could be obtained and the relationship between the mismatch repair status and OS was not investigated.

Owing to the higher risk of heterochronous PM recurrence, we focused more on S-PM and *KRAS*/*BRAF*, and found that *BRAF* was significantly associated with OS in patients with S-PM. Meanwhile, we found that patients with *mutBRAF* had worse PFS than those with mut*KRAS*. Moreover, the nomogram constructed in this study is probably superior to existing predictive models that do not include any tumor gene status [38–41]. Furthermore, this nomogram exhibits a good predictive ability, with a high C-index. Nonetheless, patients carrying mut*BRAF* should be more cautious while opting for CRS.

The present study has some limitations. First, the retrospective nature and single-center data used in this study may have contributed to certain biases. Thus, prospective multicenter studies should be conducted to validate the present results. Second, some patients were not included in the study because of the deletion of their genetic statuses. Third, we obtained the *BRAF*/*KRAS* statuses from the patients’ pathology reports, which may have contained some errors. For financial reasons, some patients were only tested for a common type of *BRAF/KRAS* and did not have their entire genome sequenced. Finally, the nomogram established was not externally validated and further studies are warranted.

## Conclusions

mut*BRAF* is an independent prognostic risk factor for S-PM in patients with CRC. In this study, we developed and validated an innovative nomogram to predict OS in patients with S-PM CRC on the basis of the *BRAF* status, which showed good discriminative ability and high accuracy in predicting the 1- and 2-year OS of patients with CRC having S-PM.
